# Development and evaluation of an attenuated *Avibacterium paragallinarum* strain as a live vaccine candidate for infectious coryza

**DOI:** 10.1186/s13567-025-01546-4

**Published:** 2025-06-09

**Authors:** Mengjiao Guo, Haonan Wang, Donghui Liu, Zongyi Bo, Chengcheng Zhang, Yantao Wu, Xiaorong Zhang

**Affiliations:** 1 Jiangsu Co-Innovation Center for Prevention of Animal Infectious Diseases and Zoonoses, College of Veterinary Medicine, Yangzhou University, Yangzhou, 225009 China; 2 Joint International Research Laboratory of Agriculture & Agri-Product Safety, Yangzhou University (JIRLAAPS), Yangzhou, 225009 China

**Keywords:** *Avibacterium paragallinarum*, attenuated strain, vaccine candidate, biofilm, pathogenicity

## Abstract

*Avibacterium paragallinarum* (*Av. paragallinarum*), the causative agent of infectious coryza, is a significant pathogen responsible for substantial economic losses in the poultry industry. Current preventive strategies rely primarily on inactivated vaccines, which have limitations such as vaccine failure and limited cross-protection between serotypes. This study aimed to develop an attenuated strain of *Av. paragallinarum* as a potential live vaccine candidate. Using the Tn5-Kan transposon, we constructed a transposon mutant library and identified a mutant strain, designated 2019/HB64-40, which harbored a disrupted *ksgA* gene encoding a critical enzyme involved in ribosomal RNA methylation. Compared with the wild-type strain, the 2019/HB64-40 strain presented significantly reduced biofilm formation, lower hemagglutination titres, and impaired growth. Pathogenicity assessments in chickens demonstrated that the mutant strain displayed significantly attenuated virulence, characterized by fewer clinical symptoms and reduced bacterial shedding. Furthermore, following challenge, all unimmunized chickens presented severe clinical signs of infectious coryza at 2 dpi, with symptoms beginning to ameliorate by 5 dpi, culminating in a mean clinical sign score of 2.1. In contrast, only one chicken (1/10) in the immunized group displayed mild facial swelling and nasal discharge, with a mean clinical sign score of 0.1. The immunized group receiving the 2019/HB64-40 strain demonstrated 90% immunoprotection, highlighting the potential of this attenuated strain as a live vaccine candidate. While cross-serotype protection was not evaluated, the results suggest effective homologous protection and colonization capacity, underscoring its promising application in the prevention and treatment of infectious coryza.

## Introduction

*Avibacterium paragallinarum* (*Av. paragallinarum*), a member of the family *Pasteurellaceae*, genus *Avibacterium*, causes infectious coryza (IC) in chickens. IC is characterized by nasal discharge, facial swelling, and conjunctivitis, leading to significant economic losses in the poultry industry worldwide due to retarded growth in growing chickens and reduced egg production in layers [1]. Strict biosecurity measures, vaccination, and antibiotics are the primary methods used to prevent and control IC. Currently, only inactivated vaccines are available, including monovalent (mostly serovar A), bivalent (serovars A and C), and trivalent inactivated vaccines (serovars A, B, and C). Three serovars, A, B, and C, are widespread and prevalent worldwide. Therefore, bivalent and inactivated trivalent inactivated IC vaccines are mainly used on poultry farms. Recently, the incidence of IC has increased in China, with cases even occurring in chickens that have been immunized with inactivated vaccines [2]. These findings suggest that some cases may be associated with vaccine failure.

There are various opinions on the reasons for vaccine failure. Some researchers have suggested that the current commercial inactivated vaccines do not offer adequate protection. It is widely believed that cross-protection between different serovars is limited. Inactivated vaccines provide protection only against the serovar strains contained in the vaccine [3–5]. The lack of ideal cross-protection within the same serovar is responsible for the ineffectiveness of inactivated vaccines. Good cross-protection is prevalent between A1-A4 in serogroup A, whereas lower cross-protection exists within some of the four serovars C1-C4 in serogroup C [6]. Although there are only B1 serovars in serogroup B, partial cross-protection has been observed between serovar B strains [7–9]. Furthermore, a previous study showed that variant strains and increased virulence lead to a reduction in the protection provided by inactivated vaccines [10]. The advantages of inactivated vaccines are simple preparation, easy storage and transport, and high safety. However, due to the presence of bacterial components such as lipopolysaccharides in inactivated whole-cell bacterial vaccines, severe side effects often occur in chickens after vaccination.

Bacterial biofilms, which are complex communities of microorganisms encased in a self-produced extracellular matrix, play a significant role in bacterial virulence and persistence. Most bacteria are capable of forming biofilms and are widespread in nature [11]. Biofilms have been shown to increase the virulence of bacteria by providing protection against the host immune system [12], facilitating the exchange of genetic material [13], and increasing resistance to antimicrobial agents [14]. Given that biofilms are often resistant to conventional antimicrobial treatments, the study of biofilm-associated infections has gained increasing attention. Strategies to combat biofilm-related infections include the development of novel antimicrobial agents, antibiofilm agents, and targeted interventions against specific biofilm-related pathways [15]. Understanding the complex interplay between biofilms and pathogenesis is crucial for the development of effective treatment strategies and the prevention of biofilm-associated infections.

Several studies have effectively harnessed the whole-genome strategy to delineate genes crucial for biofilm formation. This has been accomplished through the application of random transposon mutagenesis in various bacterial pathogens, including *Escherichia coli* O157:H7 [16], *Salmonella enteritidis* [17], and *Staphylococcus aureus* [18]. However, few studies on the biofilm formation of *Av. paragallinarum*. On the basis of the natural transformation method established in our previous study for *Av. paragallinarum* [19], this study utilized transposon technology to generate a mutant strain with impaired biofilm formation. Furthermore, we evaluated the potential of this mutant strain as a live vaccine candidate, laying the groundwork for the development of an attenuated live vaccine against *Av. paragallinarum*.

## Materials and methods

### Bacterial strains, culture conditions, chickens

*Av. paragallinarum* 2019/HB64 (serotype A) was isolated from chickens affected by IC and cultured on tryptic soy agar (TSA) or tryptic soy broth (TSB) supplemented with 10% fetal bovine serum and 0.0025% nicotinamide adenine dinucleotide [2].

A transposon mutant library of *Av. paragallinarum* 2019/HB64 was constructed using the Tn5-Kan transposon. To select for the growth of bacterial strains, kanamycin (10 mg/mL) was added to the culture medium.

Twenty-eight-day-old specific pathogen-free (SPF) White Leghorn chickens were acquired from Boehringer-Ingelheim (Beijing, China). All experimental procedures were conducted in accordance with the guidelines and ethical approval provided by the Committee on the Ethics of Animal Experiments at Yangzhou University.

### Biofilm quantification and growth curve

Overnight cultures of *Av. paragallinarum* were inoculated into TSB at a ratio of 1:100 in 96-well microplate and cultured at 37 °C for 24 h. Quantification of biofilm production was performed using crystal violet staining, as described previously [20]. In brief, the cut-off optical density (ODc) was defined as three standard deviations above the mean OD of the negative control. The biofilm production capabilities of the strains were categorized as follows: OD ≤ ODc indicated no biofilm production, ODc < OD ≤ 2 × ODc denoted weak biofilm production, 2 × ODc < OD ≤ 4 × ODc represented moderate biofilm production, and OD > 4 × ODc was classified as strong biofilm production.

Overnight cultures of *Av. paragallinarum* were inoculated into TSB at a ratio of 1:100 and cultured at 37 °C for 24 h. The OD_600_ value of the bacterial suspension was measured every hour using a spectrophotometer to plot the growth curve of the strain. All experiments were performed in triplicate, and the mean values of the results were recorded.

### Hemagglutination titre

The *Av. paragallinarum* were grown in TSB at 37 °C for 16 h. Subsequently, the bacterial suspension was centrifuged and washed 3 times with phosphate-buffered saline (PBS), and the bacteria were then resuspended in PBS as antigens. Fixed chicken erythrocytes treated with glutaraldehyde were employed in a hemagglutination assay (HA). The highest antigen dilution that elicits complete agglutination of formaldehyde-treated chicken erythrocytes was defined as the HA titre for the corresponding strain.

### Adhesion test

To investigate the adhesion properties of the mutant strain, DF-1 cells were cultured in 24-well plates until they reached approximately 80% confluence. *Av. paragallinarum* strains were added to DF-1 cells and incubated for 1 h (MOI = 100:1). DF-1 cells were gently washed with PBS to remove nonadherent bacteria. DF-1 cells were then lysed with 1% Triton X-100 solution for 20 min, and the lysate was spread on TSA for counting.

### Identification of the transposon insertion site of *Av. paragallinarum* mutant 2019/HB64-40

The site of Tn5-Kan insertion was determined using a chromosome walking kit (TaKaRa, Dalian, China) with three specific primers (SP1, SP2, and SP3) according to the manufacturer’s instructions. The primer sequences are shown in Table 1. The sequence of the identified gene was analysed using NCBI BLAST to identify homologous sequences and putative functions. Functional analyses of the mutated genes were performed via the PSORT website and the EggNOG website.

**Table 1:** **Specific primers used in this study.**

Primer name	Sequence (5ʹ–3ʹ)
SPF1	GCTTGCCGAATATCATGGTGGA
SPF2	ATTCGCAGCGCATCGCCTTCTATC
SPF3	CGCCCAACCTGCCATCACGAGATTT
APG-F	AGCTTGCTCTACCGCACAAT
APG-R	CTGGCTTCTTGCACCTGAAT

### Pathogenicity of the* Av. paragallinarum* mutant 2019/HB64-40

To determine the pathogenicity of the 2019/HB64-40 mutant, 45 chickens were randomly divided into 3 groups and maintained in negative-pressure isolators. The pathogenicity experiment was conducted by inoculating the infraorbital sinus with 0.1 mL of *Av. paragallinarum* mutant 2019/HB64-40 or 2019/HB64 bacterial suspensions (10^7^ cfu/mL). The control group received an inoculation of 0.2 mL of PBS. The process of the pathogenicity experiment is shown in Figure 2A. Clinical signs were monitored and recorded following challenge with *Av. paragallinarum* according to a previous study [21]. The clinical sign scoring criteria were divided into four categories: 0: absence of clinical symptoms; 1: mild facial swelling and minimal nasal discharge; 2: moderate facial swelling and noticeable nasal discharge; and 3: severe facial swelling, copious nasal discharge, and lacrimation. At 3, 7, and 14 days post-infection (dpi), five chickens from each group were randomly selected and euthanized. Lesions of the infraorbital sinus and trachea were observed.

At 3, 7, and 14 dpi, nasal swabs were collected for *Av. paragallinarum* quantification. DNA extraction was performed using the Bacteria Genomic DNA Kit (CWBIO, Beijing, China) following the manufacturer’s protocol. The primers used for the detection of *Av. paragallinarum* have been previously described and are provided in Table 1 [22]. Quantitative real-time PCR (qRT‒PCR) was conducted with TransStartR Tip Green qPCR SuperMix (TransGen Biotech Co., Ltd., Beijing, China). The qRT‒PCR procedure was as follows: 94 °C for 30 s, followed by 40 cycles of 94 °C for 5 s and 60 °C for 34 s. Subsequently, a dissociation curve analysis was performed to assess the specificity of the amplification products.

### Immune protection test

Thirty chickens were randomly divided into 3 groups and maintained in separate negative-pressure isolators. The chickens in the vaccinated group were inoculated with 0.1 mL of *Av. paragallinarum* mutant 2019/HB64-40 (10^6^ cfu/mL) via the intranasal and intraocular routes, whereas chickens in the challenge group were inoculated with PBS. Three weeks after vaccination, the chickens in the vaccinated group and challenge groups were challenged by infraorbital sinus inoculation with 0.1 mL of the *Av. paragallinarum* 2019/HB64 bacterial suspension (10^7^ cfu/mL). The negative control group did not receive any treatment. The clinical sign scores were monitored and recorded after challenge with *Av. paragallinarum*. All chickens were weighed before and at 7 dpi. Nasal swabs were collected for bacterial shedding at 3, 5 and 7 dpi. At 7 dpi, all chickens were euthanized. Lesions of the infraorbital sinus and trachea were observed (Figure 3A).

The infraorbital sinus and trachea were collected and fixed in 4% paraformaldehyde solution for 24 h. The fixed tissues were then processed for paraffin embedding, sectioned into 5 μm slices using a microtome, and mounted on glass slides. The sections were deparaffinized, rehydrated, and stained with hematoxylin and eosin (H&E). The microscopic lesions were then observed using a digital slice scanner (NanoZoomer NDP.view2).

### Statistical analysis

Statistical analyses were performed with SPSS version 23.0 (SPSS Inc., Chicago, IL, USA). The nonparametric Mann–Whitney test was used to analyse significant differences. A statistically significant difference was considered at *p* < 0.05.

## Results

### Characterization of the *Av. paragallinarum* mutant strain 2019/HB64-40

**Figure 1:**
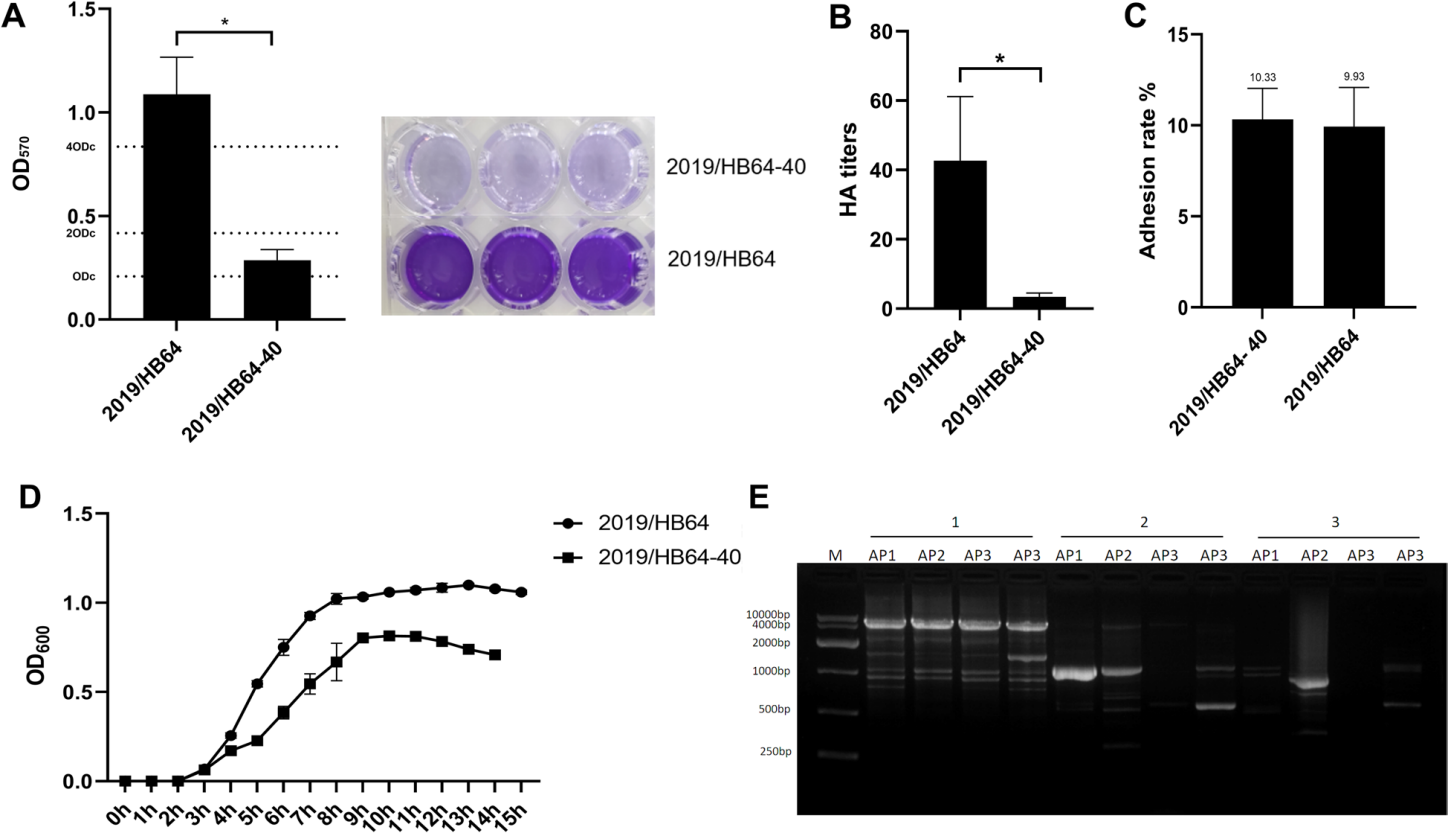
**Characterization of the** ***Av. paragallinarum*** **mutant strain 2019/HB64-40**. **A** Biofilm quantification of the 2019/HB64-40 mutant strain and the wild-type strain. The crystal violet staining method was used to determine biofilm formation ability. **B** HA titre of the mutant strain 2019/HB64-40 and the wild-type strain agglutinating formaldehyde-treated chicken erythrocytes. **C** Adhesion rates of the mutant strain 2019/HB64-40 and the wild-type strain to DF-1 cells. **D** Growth curves of the 2019/HB64-40 mutant strain and the wild-type strain. The OD_600_ values were measured every hour by using a spectrophotometer. **E** Chromosome walking PCR of strain 2019/HB64-40. M: DL10000 DNA marker; 1: amplification products of primer SPF1 with parsimony primers AP1, AP2, AP3, and AP4; 2: amplification products of primer SPF2 with parsimony primers AP1, AP2, AP3, and AP4; 3: amplification products of primer SPF3 with parsimony primers AP1, AP2, AP3, and AP4, respectively.

*Av. paragallinarum* mutant strain 2019/HB64-40 was screened from the transposon random mutation library and exhibited a significantly reduced ability to form biofilms compared to the wild-type strain (*p* < 0.05), indicating weak biofilm formation (Figure 1A). The results of the HA assay revealed that the HA titre of the wild-type strain 2019/HB64 was 2^5^, whereas that of the mutant strain 2019/HB64-40 was only 2^1^ (Figure 1B). However, the adhesion ability of 2019/HB64-40 to DF-1 cells was not significantly different from that of the 2019/HB64 strain (Figure 1C). The growth curves of the wild-type and mutant strains were further determined, and the results revealed that both strains entered the logarithmic growth phase after 2 h, but their growth rate was slower than that of the wild-type strain. The OD_600_ value of 2019/HB64 reached approximately 1, whereas that of the mutant strain 2019/HB64-40 reached only approximately 0.8 and quickly entered the decline phase from the plateau phase (Figure 1D).

### Identification of genes disrupted by Tn5-Kan insertion

In the third round of PCR, only a single band was amplified in the lane of the AP2 primer (Figure 1E), and the transposon insertion site sequence was sequenced. According to the sequence alignment results of BLAST in NCBI, the mutated gene in 2019/HB64-40 was identified as the *ksgA* gene. The *ksgA* gene, located in the cytoplasm, encodes 16S rRNA adenine dimethyltransferase, which catalyzes the methylation of two adenosines (A1518 and A1519) within the 3’-proximal helix of the small subunit rRNA. *KsgA* plays a pivotal role in the assembly of the 30S ribosomal subunit, serving as a crucial checkpoint in this process.

### Pathogenicity of the *Av. paragallinarum* mutant in chickens

**Figure 2:**
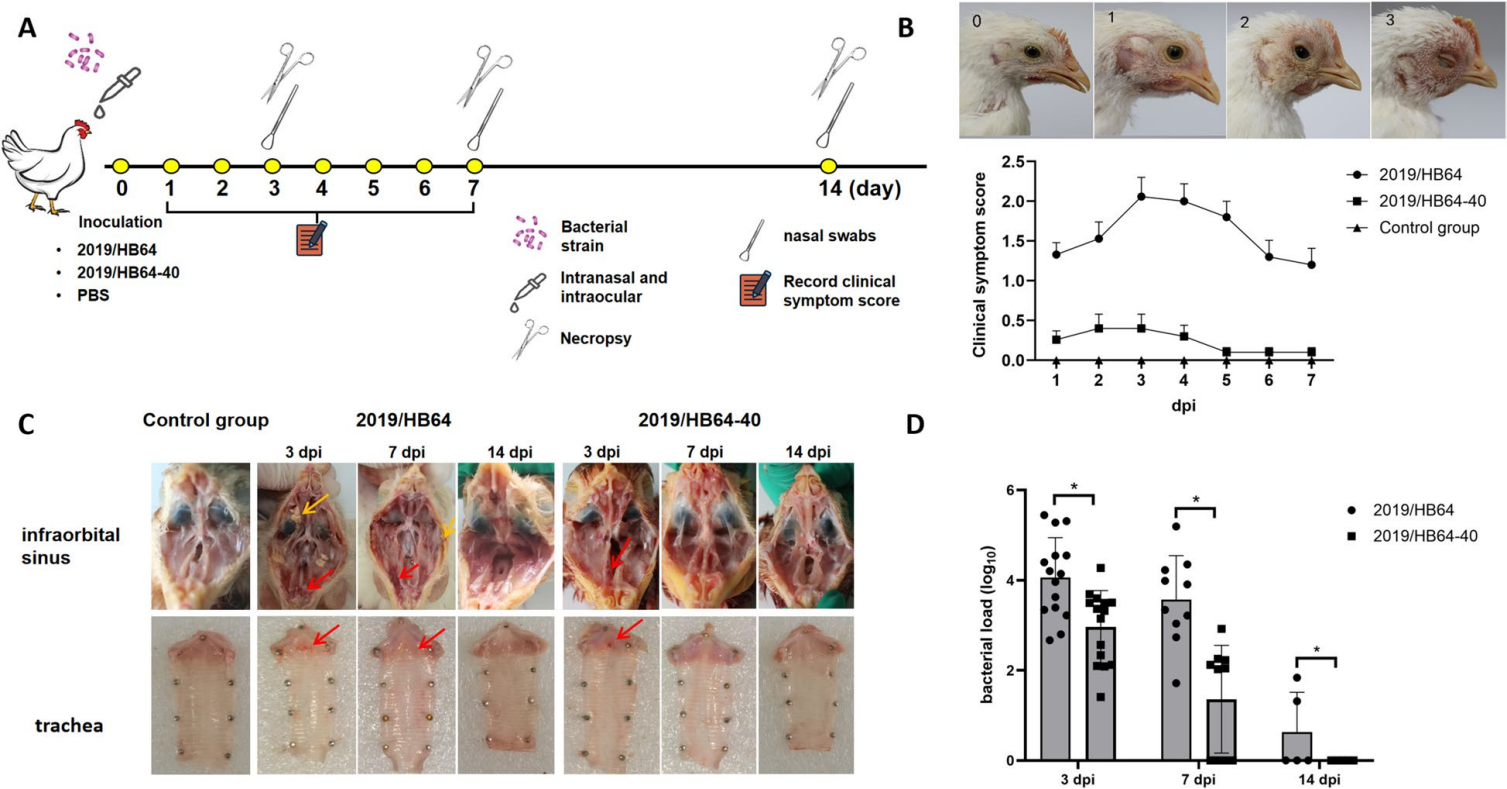
**Pathogenicity of the** ***Av. paragallinarum*** **mutant strain 2019/HB64-40**. **A** Schematic diagram of animal experiment implementation. **B** Clinical symptom scoring criteria corresponding to clinical manifestations and clinical symptom scores. **C** Pathologic changes in chickens at 3, 7, and 14 days after inoculation with 0.1 mL of *Av. paragallinarum* mutant 2019/HB64-40 or 2019/HB64. The areas indicated by yellow arrows represent caseous exudate, and red arrows indicate hemorrhage. **D** Bacterial shedding in chickens after challenge with *Av. paragallinarum* mutant 2019/HB64-40 or 2019/HB64. Nasal swab samples were collected at 3, 7, and 14 days post-inoculation.

At 3 dpi, the incidence rate of chickens challenged with the wild-type strain 2019/HB64 was 100%. The clinical manifestations included depression, severe facial swelling and copious secretions around the eyes and nose. However, only 4/15 chickens challenged with the mutant strain 2019/HB64-40 exhibited clinical symptoms of IC, primarily mild facial swelling. The clinical symptoms of all chickens in the 2019/HB64-40 group had completely resolved at 7 dpi, while those in the wild-type strain challenge group still had an average clinical symptom score of 1.2 (Figure 2B).

The necropsy results revealed that in the 2019/HB64 challenge group, yellow caseous exudate was observed in the infraorbital sinus, with the nasal cavity filled with mucus and petechial hemorrhages visible in the tracheal larynx at 3 dpi. At 7 dpi, the lesions had mitigated, although some chickens still presented small amounts of caseous exudate and petechial hemorrhages. Complete recovery was achieved at 14 dpi. In the 2019/HB64-40 challenge group, mild facial swelling was noted in 4/15 chickens at 3 dpi, but no caseous exudate was detected. Moreover, all symptoms had completely subsided, and no pathological changes were observed at 7 dpi. The control group exhibited no lesions (Figure 2C).

The bacterial shedding results revealed that the bacterial shedding in the 2019/HB64-40 challenge group was significantly lower than that in the 2019/HB64 challenge group at 3 dpi and 7 dpi (*p* < 0.05). The highest bacterial shedding in the 2019/HB64 challenge group reached 2.78 × 10^5^ copies, with an average of 5.45 × 10^4^ copies, whereas the 2019/HB64-40 challenge group had a maximum of only 1.87 × 10^4^ copies, with an average of 2.92 × 10^3^. At 7 dpi, bacterial shedding in the 2019/HB64-40 challenge group significantly decreased, whereas the 2019/HB64 challenge group maintained a shedding copy number of 2.17 × 10^4^. At 14 dpi, the 2019/HB64 group presented only a few dozen copies of bacterial shedding, and no bacterial shedding was detected in the 2019/HB64-40 group (Figure 2D). These results indicate that the pathogenicity of strain 2019/HB64-40 is significantly lower than that of the wild-type strain.

### Protective efficacy of the attenuated live vaccine candidate strain 2019/HB64-40

**Figure 3:**
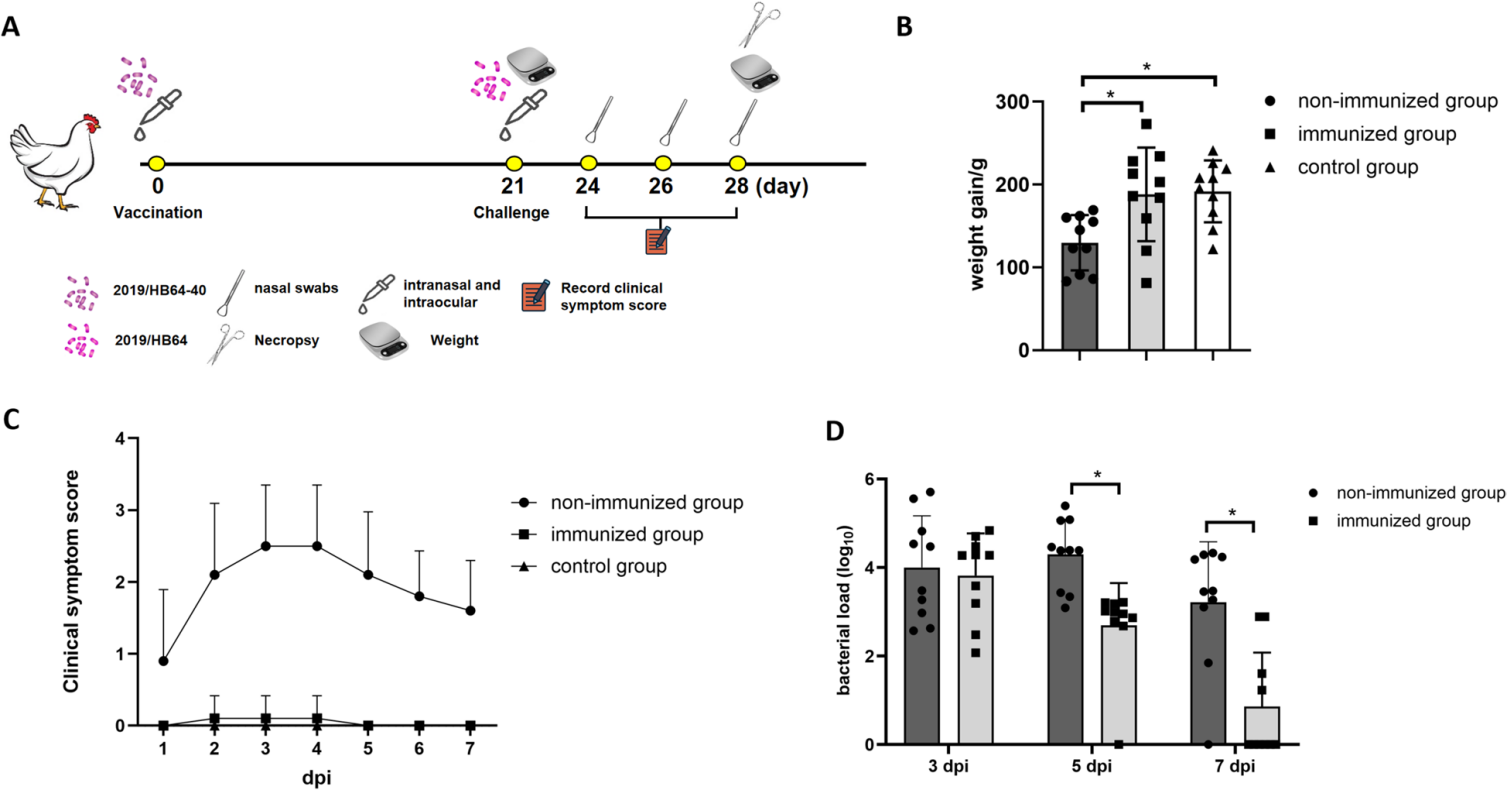
**Protective efficacy of the 2019/HB64-40 strain.** **A** Schematic diagram of the animal experiment implementation. **B** Weight gain of chickens after challenge with *Av. paragallinarum* 2019/HB64. All chickens were weighed before and 7 days after challenge. **C** Clinical symptom scores of chickens after challenge with 2019/HB64. **D** Bacterial shedding in chickens after challenge with 2019/HB64. The nasal swab samples were collected at 3, 5, and 7 dpi.

The clinical symptom scoring results revealed that all chickens in the non-immunized group presented clinical symptoms of IC within 3 dpi. As shown in Figure 3B, there was no significant difference in weight gain between the 2019/HB64-40-immunized group and the control group after challenge. However, the weight gain in the non-immunized group was significantly lower than that in the control group and the immunized group (*p* < 0.05). All chickens in the non-immunized group presented depression, loss of appetite, severe facial swelling, and the presence of secretions in the eyes and nose, with an average clinical symptom score of 2.5. In the *Av. paragallinarum* 2019/HB64-40-immunized group, only 1/10 of the chickens developed mild IC symptoms at 3 dpi, with an average clinical symptom score of 0.1, and the affected chickens quickly recovered by 5 dpi (Figure 3C).

At 3 dpi, both the immunized and non-immunized groups presented high levels of bacterial shedding, with no significant difference observed. At 5 dpi, the average bacterial shedding copy number in the non-immunized group was 5.91 × 10^4^, whereas the immunized group had a significantly lower shedding copy number, averaging only 9.50 × 10^2^ (*p* < 0.05). At 7 dpi, most chickens in the immunized group showed no detectable bacterial shedding, while the average shedding in the non-immunized group was still 8.22 × 10^3^ (Figure 3D). Therefore, immunization with 2019/HB64-40 can effectively reduce respiratory tract bacterial shedding in *Av. paragallinarum*.

**Figure 4:**
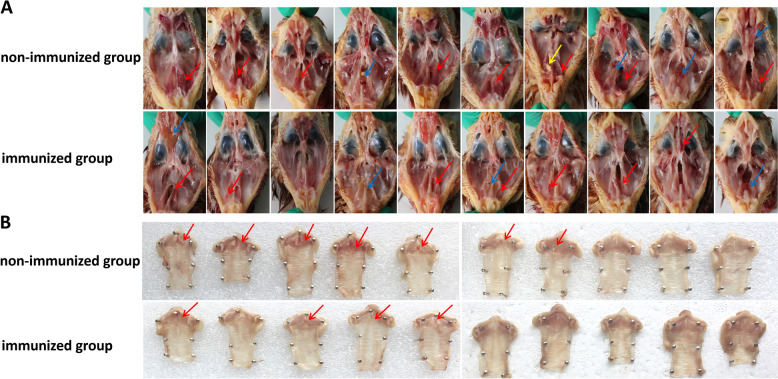
**Gross lesions in chickens at 7 days after challenge with** ***Av. paragallinarum.*** **A** Pathologic changes in the infraorbital sinus. **B** Pathologic changes in the trachea. The areas indicated by yellow arrows represent caseous exudate, blue arrows represent mucus, and red arrows indicate haemorrhage.

At 7 dpi, necropsy revealed that all chickens in the non-immunized group exhibited facial swelling. One chicken had yellow caseous exudate in the infraorbital sinus, and 8/10 chickens experienced bleeding, with 5 of them experiencing severe bleeding. Mucus was observed in the nasal cavity of 4/10 chickens, and petechial bleeding was obvious in the trachea and throat of 7/10 chickens. In the 2019/HB64-40 group, no yellow caseous exudate was found in the infraorbital sinus. Slight bleeding in the infraorbital sinus was observed in 7/10 chickens, mucus in the nasal cavity in 4/10 chickens, and slight bleeding in the trachea and throat in 4/10 chickens (Figure 4 and Table 2).

**Table 2:** **Gross lesions of chickens after challenge with** ***Av. paragallinarum.***

Groups	Gross lesions		1	2	3	4	5	6	7	8	9	10	Total
Non-immunized group		Hemorrhagic	++	++	+	−	++	++	+	++	−	+	8/10
	Infraorbital sinus	Mucus	−	−	−	+	−	−	−	+	+	+	4/10
		Caseous exudate	−	−	−	−	−	−	+	−	−	−	1/10
	Trachea	Hemorrhagic	++	++	++	++	++	+	+	−	−	−	7/10
Immunized group		Hemorrhagic	+	+	−	−	+	+	+	+	+	−	7/10
	Infraorbital sinus	Mucus	+	−	−	+	−	+	−	−	−	+	4/10
		Caseous exudate	−	−	−	−	−	−	−	−	−	−	0/10
	Trachea	Hemorrhagic	+	−	+	+	+	−	−	−	−	−	4/10
Control group		Hemorrhagic	−	−	−	−	−	−	−	−	−	−	0/10
	Infraorbital sinus	Mucus	−	−	−	−	−	−	−	−	−	−	0/10
		Caseous exudate	−	−	−	−	−	−	−	−	−	−	0/10
	Trachea	Hemorrhagic	−	−	−	−	−	−	−	−	−	−	0/10

“−” indicates no lesions; “+” indicates mild histological changes; “++” indicates severe histological changes.

**Figure 5:**
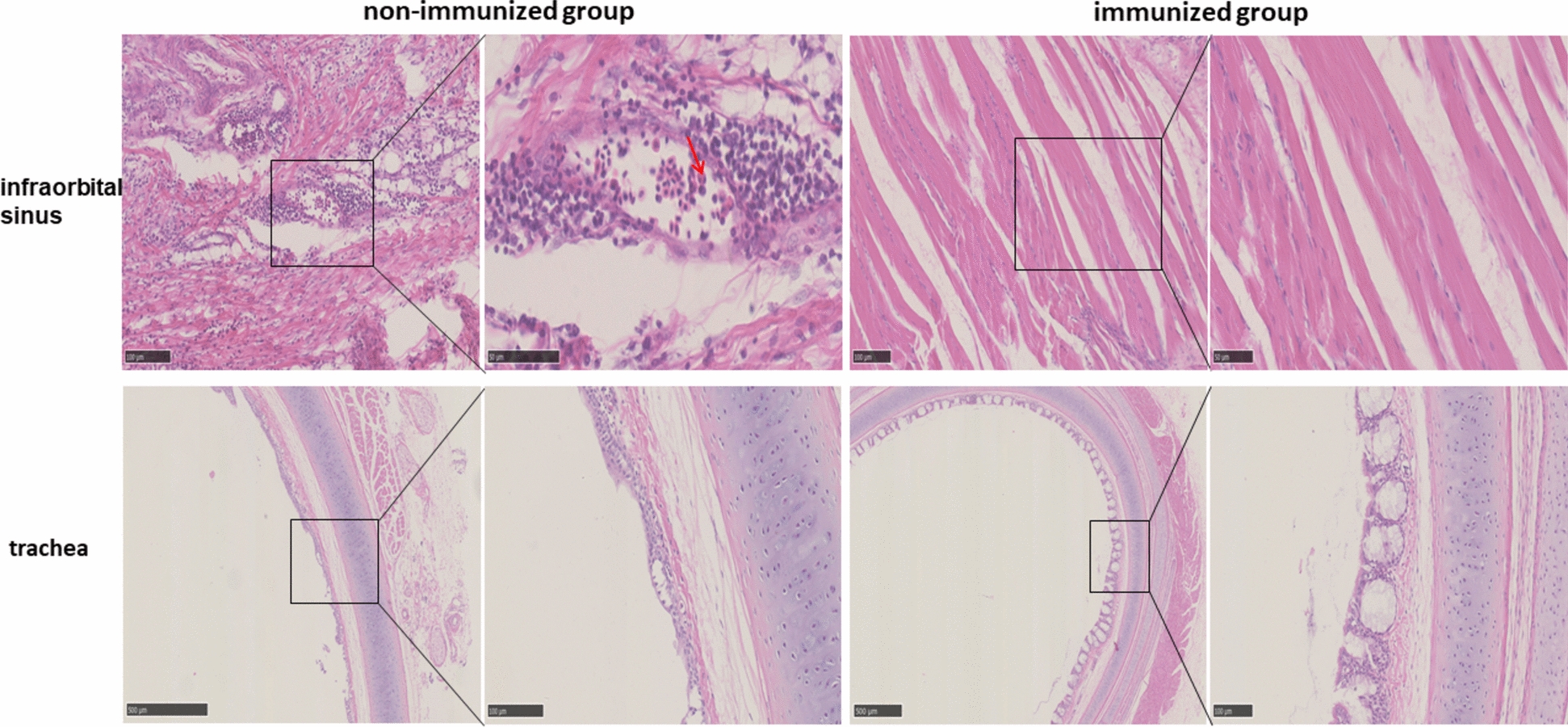
**Pathological changes in chickens after challenge with** ***Av. paragallinarum.*** Edema, tissue looseness, and hemorrhage were observed in the infraorbital sinus of non-immunized group. The infiltration of macrophages and heterophilic granulocytes is indicated by red arrows. Tracheal cilia exfoliation and edema of the lamina propria mucosa were observed in non-immunized chickens. Only partial adhesion of tracheal cilia was observed in the immunized chickens.

As shown in Figure 5, histopathological analysis revealed significant pathological changes in the non-immunized group. Specifically, oedema and tissue looseness were observed in the infraorbital sinus. Additionally, hemorrhage and infiltration of macrophages and heterophilic granulocytes were noted in the infraorbital sinus. In the trachea, cilia exfoliation and edema of the lamina propria mucosa were evident in non-immunized chickens. In contrast, only partial adhesion of tracheal cilia was observed in the immunized chickens, suggesting a potential protective effect of immunization against these pathological changes.

## Discussion

The vaccines currently used to prevent and control IC are whole-cell inactivated vaccines. Whole-cell inactivated vaccines contain lipopolysaccharides and bacterial proteins that can cause adverse reactions, and most vaccines require multiple immunizations to achieve a high level of humoral immunity and effective immunoprotection. Compared with inactivated vaccines and subunit vaccines, live attenuated vaccines offer several advantages, including an administration route similar to natural infection and the ability to induce a comprehensive immune response, encompassing humoral, cellular, and mucosal immunity, demonstrating significant potential for application. The use of transposons for random mutagenesis is a powerful tool for investigating the functional genes of target strains and screening attenuated strains. Therefore, in this study, the *Av. paragallinarum* wild-type strain 2019/HB64, which exhibits high natural transformation efficiency, was used as the parental strain, and a random mutation library was constructed using transposons. The biological properties of the mutant strains, such as biofilm formation ability, were screened in the mutation library, and a potential candidate strain was selected for further evaluation of pathogenicity and immune protection.

Bacterial biofilms are associated with bacterial resistance to adverse environments and the development of drug resistance, making them an important indicator of bacterial virulence [23]. The hemagglutination ability of *Av. paragallinarum* has also been proven to be closely related to its virulence and serotyping ability [24, 25]. On the basis of the transposon random mutagenesis library, a mutant strain, 2019/HB64-40, with significantly reduced biofilm formation and hemagglutination ability was screened. Using chromosome walking technology, the transposon insertion site was identified as the *ksgA* gene, which encodes ribosomal RNA small subunit methyltransferase A. This protein primarily acts on the 30S subunit of ribosomes by dimethylating two adjacent adenine residues in a conserved hairpin loop near the 3ʹ end of 16S rRNA [26]. In addition, the mutant strain 2019/HB64-40 exhibited impaired growth performance. Unlike in wild-type bacteria, knocking out the *ksgA* gene often leads to unfavourable growth. This growth impairment has been observed across various organisms, including *Escherichia coli* [26], *Mycobacterium tuberculosis* [27], and *Chlamydia trachomatis* [28], where the absence or alteration of *ksgA* results in either reduced growth rates or an inability to effectively compete against wild-type strains. The pathogenicity evaluation results revealed a significant reduction in pathogenicity compared with that of the wild-type strain. The deletion of *ksgA* has been demonstrated to attenuate the virulence of *Yersinia pseudotuberculosis* [29]. Similarly, in the plant pathogen *Erwinia amylovora*, inactivating mutations of *ksgA* have been linked to a reduction in virulence [30]. These observations highlight the crucial role of *KsgA* in maintaining optimal growth and virulence in bacteria. In addition to pathogenicity assessment, we also conducted stability experiments on 20 consecutive passages in antibiotic-free culture medium. The Tn5-ksgA mutation is stable, and weak biofilm production remains unchanged (data not shown). While in vitro passage experiments demonstrated stable genetic attenuation, the behavior of live vaccine strains in host environments requires careful evaluation. Future studies will monitor the recovery and genetic stability of the mutation strain during prolonged and multiple in vivo passages in chickens.

Several studies have constructed attenuated strains of *Av. paragallinarum* through gene editing. For example, a heme utilization mutant strain of *Av. paragallinarum* exhibits significantly reduced growth under iron-restricted conditions, compromised acid stress tolerance, and decreased virulence [31]. The acapsular mutants of *Av. paragallinarum* exhibited an increased ability to adhere to DF-1 cells and form biofilms on abiotic surfaces but decreased virulence [32]. Additionally, F17-like fimbriae family A-deficient mutants of *Av. paragallinarum* exhibit attenuated virulence [33]. However, none of these studies have successfully developed and applied an attenuated *Av. paragallinarum* strains as vaccine candidates.

Following challenge, all unimmunized chickens presented severe clinical signs of IC. In contrast, only one chicken in the immunized group displayed mild facial swelling and nasal discharge, which corresponded to a 90% rate of immunoprotection. Necropsy revealed the absence of caseous exudate in the infraorbital sinus of the immunized group, whereas the non-immunized group displayed a significant presence of yellow caseous exudate in the facial subcutaneous tissue and infraorbital sinus. *Av. paragallinarum*, which targets the upper respiratory tract, primarily colonizes this area and triggers corresponding pathological changes. The clinical symptom score is an important index for evaluating the immune protection conferred by a vaccine, and bacterial shedding in the respiratory tract reflects the persistence of infection and the potential for horizontal transmission. Gallardo et al. assessed the efficacy of two commercial vaccines against *Av. paragallinarum* isolates, and experiments involving SPF chickens demonstrated that vaccination with two doses of a commercial vaccine significantly reduced bacterial shedding post-challenge [34]. In this study, the group immunized with the 2019/HB64-40 strain also presented a significant decrease in bacterial shedding in the upper respiratory tract. These results indicate that the attenuated strain 2019/HB64-40 plays a significant role in alleviating clinical symptoms and shortening the duration of infection, thereby providing good immune protection. This study demonstrated the potential of 2019/HB64-40 as a live vaccine candidate. However, its comparative efficacy with that of commercial inactivated vaccines remains to be evaluated. Current inactivated vaccines face challenges in covering diverse serovars and emerging variants, and the potential of live attenuated candidates to address this gap remains untested. Therefore, another limitation of this study is the absence of a cross-serotype protection evaluation. Future studies will focus on cross-serovar protection, wild-type strain competition, immunization procedures, and duration of immunity. These findings elucidate the immune protection mechanism of the live 2019/HB64-40 vaccine candidate.

In conclusion, an attenuated strain was constructed and screened using transposons in this study. The attenuated strain 2019/HB64-40 not only demonstrated a diminished ability to form biofilms and hemagglutinate but also elicited a robust immune response, leading to a significant reduction in bacterial shedding and clinical symptoms post-challenge. The strain exhibits low pathogenicity and good safety, with a protection rate of up to 90% in chickens. This study provides valuable insights for the development of novel live attenuated vaccines against *Av. paragallinarum*.

## Data Availability

The datasets supporting the conclusions of this article are available from the corresponding author upon reasonable request.

## References

[1] Blackall PJ, Soriano‐Vargas E (2020) Infectious coryza and related bacterial infections. In: David E. Swayne (eds) Diseases of Poultry, 13^th^ Edition pp: 890–906

[2] Guo M, Chen X, Zhang H, Liu D, Wu Y, Zhang X (2022) Isolation, serovar identification, and antimicrobial susceptibility of *Avibacterium paragallinarum* from chickens in China from 2019 to 2020. Vet Sci 1:27 10.3390/vetsci9010027 PMC8781767 35051111

[3] Blackall PJ, Reid GG (1987) Further efficacy studies on inactivated, aluminum-hydroxide-adsorbed vaccines against infectious coryza. Avian Dis 31:527–532 2960312

[4] Rimler RB, Davis RB, Page RK (1977) Infectious coryza: cross-protection studies, using seven strains of *Haemophilus gallinarum*. Am J Vet Res 38:1587–1589 931140

[5] Kume K, Sawata A, Nakase Y (1980) Immunologic relationship between Page’s and Sawata’s serotype strains of *Haemophilus paragallinarum*. Am J Vet Res 41:757–760 7406296

[6] Soriano EV, Garduño ML, Téllez G, Rosas PF, Suárez-Güemes F, Blackall PJ (2004) Cross-protection study of the nine serovars of *Haemophilus paragallinarum* in the Kume haemagglutinin scheme. Avian Pathol 33:506–511 15545030 10.1080/03079450400003502

[7] Yamaguchi T, Blackall PJ, Takigami S, Iritani Y, Hayashi Y (1991) Immunogenicity of *Haemophilus paragallinarum* serovar B strains. Avian Dis 35:965–968 1838478

[8] Morales-Erasto V, Fernández-Rosas P, Negrete-Abascal E, Salazar-García F, Blackall PJ, Soriano-Vargas E (2014) Genotyping, pathogenicity, and immunogenicity of *Avibacterium paragallinarum* serovar B-1 isolates from the Americas. Avian Dis 58:293–296 25055635 10.1637/10693-101513-ResNote.1

[9] Jacobs AA, van den Berg K, Malo A (2003) Efficacy of a new tetravalent coryza vaccine against emerging variant type B strains. Avian Pathol 32:265–269 12850915 10.1080/0307945031000097859

[10] Xu Y, Cheng J, Huang X, Xu M, Feng J, Liu C, Zhang G (2019) Characterization of emergent *Avibacterium paragallinarum* strains and the protection conferred by infectious coryza vaccines against them in China. Poult Sci 98:6463–6471 31801310 10.3382/ps/pez531 PMC8913951

[11] Hall-Stoodley L, Costerton JW, Stoodley P (2004) Bacterial biofilms: from the natural environment to infectious diseases. Nat Rev Microbiol 2:95–108 15040259 10.1038/nrmicro821

[12] Vidakovic L, Mikhaleva S, Jeckel H, Nisnevich V, Strenger K, Neuhaus K, Raveendran K, Ben-Moshe NB, Aznaourova M, Nosho K, Drescher A, Schmeck B, Schulte LN, Persat A, Avraham R, Drescher K (2023) Biofilm formation on human immune cells is a multicellular predation strategy of *Vibrio cholerae*. Cell 186:2690-2704.e20 37295405 10.1016/j.cell.2023.05.008 PMC10256282

[13] Balcázar JL, Subirats J, Borrego CM (2015) The role of biofilms as environmental reservoirs of antibiotic resistance. Front Microbiol 6:1216 26583011 10.3389/fmicb.2015.01216 PMC4628128

[14] Hall CW, Mah TF (2017) Molecular mechanisms of biofilm-based antibiotic resistance and tolerance in pathogenic bacteria. FEMS Microbiol Rev 41:276–301 28369412 10.1093/femsre/fux010

[15] Yan J, Bassler BL (2019) Surviving as a community: antibiotic tolerance and persistence in bacterial biofilms. Cell Host Microbe 26:15–21 31295420 10.1016/j.chom.2019.06.002 PMC6629468

[16] Puttamreddy S, Cornick NA, Minion FC (2010) Genome-wide transposon mutagenesis reveals a role for pO157 genes in biofilm development in *Escherichia coli* O157:H7 EDL933. Infect Immun 78:2377–2384 20351142 10.1128/IAI.00156-10 PMC2876562

[17] Solano C, García B, Valle J, Berasain C, Ghigo JM, Gamazo C, Lasa I (2002) Genetic analysis of *Salmonella enteritidis* biofilm formation: critical role of cellulose. Mol Microbiol 43:793–808 11929533 10.1046/j.1365-2958.2002.02802.x

[18] Boles BR, Thoendel M, Roth AJ, Horswill AR (2010) Identification of genes involved in polysaccharide-independent *Staphylococcus aureus* biofilm formation. PLoS One 5:e10146 20418950 10.1371/journal.pone.0010146 PMC2854687

[19] Liu D, Zhang H, Tan H, Jin Y, Zhang C, Bo Z, Zhang X, Guo M, Wu Y (2023) Basic characterization of natural transformation in *Avibacterium paragallinarum*. Microbiol Spectr 11:e0520922 37212663 10.1128/spectrum.05209-22 PMC10269479

[20] Stepanović S, Cirković I, Ranin L, Svabić-Vlahović M (2004) Biofilm formation by *Salmonella* spp. and *Listeria monocytogenes* on plastic surface. Lett Appl Microbiol 38:428–432 15059216 10.1111/j.1472-765X.2004.01513.x

[21] Bragg R (2002) Virulence of South African isolates of *Haemophilus paragallinarum*. Part 1: NAD-dependent field isolates. Onderstepoort J Vet Res 69:163–169 12234003

[22] Wen S, Chen X, Xu F, Sun H (2016) Validation of reference genes for real-time quantitative PCR (qPCR)analysis of *Avibacterium paragallinarum*. PLoS One 11:e0167736 27942007 10.1371/journal.pone.0167736 PMC5152862

[23] Schilcher K, Horswill AR (2020) Staphylococcal biofilm development: structure, regulation, and treatment strategies. Microbiol Mol Biol Rev 3:e00026-19 10.1128/MMBR.00026-19 PMC7430342 32792334

[24] Yamaguchi T, Kobayashi M, Masaki S, Iritani Y (1993) Isolation and characterization of a *Haemophilus paragallinarum* mutant that lacks a hemagglutinating antigen. Avian Dis 37:970–976 8141756

[25] Wang YP, Hsieh MK, Tan DH, Shien JH, Ou SC, Chen CF, Chang PC (2014) The haemagglutinin of *Avibacterium paragallinarum* is a trimeric autotransporter adhesin that confers haemagglutination, cell adherence and biofilm formation activities. Vet Microbiol 174:474–482 25465664 10.1016/j.vetmic.2014.10.013

[26] Connolly K, Rife JP, Culver G (2008) Mechanistic insight into the ribosome biogenesis functions of the ancient protein KsgA. Mol Microbiol 70:1062–1075 18990185 10.1111/j.1365-2958.2008.06485.x PMC2709978

[27] Tufariello JM, Jacobs WR Jr, Chan J (2004) Individual *Mycobacterium tuberculosis* resuscitation-promoting factor homologues are dispensable for growth in vitro and in vivo. Infect Immun 72:515–526 14688133 10.1128/IAI.72.1.515-526.2004 PMC343985

[28] Binet R, Maurelli AT (2009) The chlamydial functional homolog of KsgA confers kasugamycin sensitivity to *Chlamydia trachomatis* and impacts bacterial fitness. BMC Microbiol 9:279 20043826 10.1186/1471-2180-9-279 PMC2807437

[29] Mecsas J, Bilis I, Falkow S (2001) Identification of attenuated *Yersinia pseudotuberculosis* strains and characterization of an orogastric infection in BALB/c mice on day 5 postinfection by signature-tagged mutagenesis. Infect Immun 69:2779–2787 11292689 10.1128/IAI.67.5.2779-2787.2001 PMC98225

[30] McGhee GC, Sundin GW (2011) Evaluation of kasugamycin for fire blight management, effect on nontarget bacteria, and assessment of kasugamycin resistance potential in *Erwinia amylovora*. Phytopathology 101:192–204 20923369 10.1094/PHYTO-04-10-0128

[31] Huo C, Jiao L, Li G, Li D, Lin W, Sun Y, Sun H (2023) HutZ is required for efficient heme utilization and contributes to the pathogenicity of *Avibacterium paragallinarum*. Microbiol Spectr 11:e0397922 37768079 10.1128/spectrum.03979-22 PMC10580934

[32] Tu TY, Hsieh MK, Tan DH, Ou SC, Shien JH, Yen TY, Chang PC (2015) Loss of the capsule increases the adherence activity but decreases the virulence of *Avibacterium paragallinarum*. Avian Dis 59:87–93 26292540 10.1637/10937-091414-reg

[33] Liu CC, Ou SC, Tan DH, Hsieh MK, Shien JH, Chang PC (2016) The fimbrial protein is a virulence factor and potential vaccine antigen of *Avibacterium paragallinarum*. Avian Dis 60:649–655 27610725 10.1637/11410-031316-Reg.1

[34] Gallardo RA, Da Silva AP, Egaña-Labrin S, Stoute S, Kern C, Zhou H, Cutler G, Corsiglia C (2020) Infectious coryza: persistence, genotyping, and vaccine testing. Avian Dis 64:157–165 32550616 10.1637/0005-2086-64.2.157

